# Impact of using ionized drinking water on growth performance and DNA damage of broiler chicks

**DOI:** 10.1007/s11250-025-04715-x

**Published:** 2025-11-18

**Authors:** Mohamed Khalil, Farid Soliman, Karim El-Sabrout, Sarah Aggag

**Affiliations:** 1 Poultry Production Department, Faculty of Agriculture, Alexandria University, Alexandria, 21545 Egypt; 2 Genetics Department, Faculty of Agriculture, Alexandria University, Alexandria, 21545 Egypt

**Keywords:** Body weight, *CaSR* mRNA, GH, Health, ORP, ROS, SCGE

## Abstract

Given the limited research on the use of water ionization devices for broiler chickens, this study aimed to explore the impact of ionized drinking water on their growth performance, oxidative status, and DNA damage. Six hundred one-day-old broiler chicks were randomly divided into two equal groups of six replicates (50 birds each). The control group (C) received tap water, and the treatment group (T) received ionized water. Water analysis for each treatment was performed. Productive traits, including water consumption and body weight, were recorded. Biochemical parameters, such as total protein, triiodothyronine (T_3_), total antioxidant capacity (TAC), and reactive oxygen species (ROS), were determined. Additionally, alkaline single-cell gel electrophoresis (SCGE) was performed to assess primary DNA damage. The results indicated that ionized water exhibited a negative oxidation-reduction potential (ORP), a characteristic often associated with antioxidant properties, and lower total dissolved solids (TDS) compared to tap water. The treated chicks showed higher water intake rates and final body weights than the control group. They also exhibited elevated levels of total protein, globulin, T_3_, TAC, and lower ROS levels than the control. Furthermore, the current results indicate that the use of ionized drinking water increases growth hormone (*GH*), insulin-like growth factor 1 (*IGF-1*), and aquaporin1 (*AQP1*) expression levels while decreasing calcium-sensing receptor (*CaSR*) and DNA damage. Consequently, the application of water ionization for broiler chicken drinking water is recommended to enhance animal productivity and health.

## Introduction

The poultry industry plays a crucial role in global food security, with broiler chickens representing a significant portion of meat production. Optimizing growth performance and ensuring the health and well-being of these birds are paramount for economic viability and sustainable practices. Various nutritional and environmental factors influence broiler growth and susceptibility to disease, and researchers are continuously exploring innovative strategies to enhance productivity and resilience (El-Sabrout et al. 2024).

Water, often considered the most critical nutrient, plays a vital role in numerous physiological processes in poultry, including nutrient transport, thermoregulation, and waste excretion. The quality and properties of drinking water can significantly impact the health and performance of broiler chicks. In recent years, interest has grown in the potential benefits of modified water sources, such as ionized water, in animal agriculture (Ebrahimi et al. 2024; Mohamed et al. 2025). Ionized water, produced through electrolysis, exhibits altered physicochemical properties compared to conventional drinking water. This process typically results in alkaline reduced water (ARW) at the cathode and acidic oxidized water (AOW) at the anode. ARW is characterized by a higher pH, negative ORP, and the presence of dissolved hydrogen gas (H_₂_). These properties have been associated with various biological effects in animal models and humans, including antioxidant activity, anti-inflammatory effects, and improved nutrient absorption (Hu et al. 2024; Mohamed et al. 2025).

In the context of broiler production, the potential benefits of ARW could include enhanced growth rates, improved feed conversion efficiency, and reduced susceptibility to oxidative stress. Oxidative stress, resulting from an imbalance between the production of ROS and the antioxidant defense system, can negatively impact cellular function, leading to DNA damage, impaired growth, and increased susceptibility to diseases in poultry. However, alkaline drinking water can improve broiler growth performance, immunity, and digestive physiology (Martínez et al. 2021). Mohamed et al. (2025) found that ionizing drinking water improved broiler chickens’ productivity, physiology, immunity, and carcass quality by increasing fluidity and reducing the ORP.

While some preliminary studies have explored the effects of modified water on poultry performance, the specific impact of ionized drinking water, particularly ARW, on the growth performance and DNA integrity of broiler chicks remains relatively underexplored. Understanding whether ARW can positively influence growth parameters and protect against DNA damage induced by environmental or metabolic stressors is crucial for determining its potential application in broiler production systems. Therefore, this study aims to investigate the impact of drinking ionized alkaline water on the growth performance and DNA integrity of broiler chicks. The findings of this research will contribute to a better understanding of the potential benefits of ionized water as a dietary intervention for enhancing broiler productivity and mitigating cellular damage, ultimately contributing to more efficient and sustainable poultry production practices.

## Materials and methods

### Animal ethics

The experimental procedures followed the ethical guidelines set by the Alexandria University Animal Care Committee (Alex. Agri. 092410306).

### Experimental design

Six hundred unsexed one-day-old *Cobb 500* broiler chicks, with an individual average weight of 39.5 ± 0.14 g, were randomly divided into two groups of 300 chicks each. Each group was further divided into six replicates of 50 birds, with each replicate housed in its own pen. The control group (C) received regular tap water, while the treatment group (T) received ionized water that was produced electrically using a local manufacturing device (WID 12 V − 30,000 Hz, Egypt), by passing electric ions through an electrode placed in a water tank (100 L) for one hour. The experiment lasted for 37 days.

### Animal husbandry

Broiler chicks were raised in an open-system house with wood-shaving bedding under consistent environmental conditions and hygienic practices. A thermal environment was maintained for the brooding period. The initial room temperature was set at 33 °C for the first 72 h, then progressively reduced by 3 °C weekly until a final temperature of 23 °C was reached, while maintaining a humidity percentage around 60%. A continuous illumination program was implemented, providing 23 h of light followed by 1 h of darkness. The routine vaccination was performed, including avian influenza (by S/C injection of inactivated vaccine at one week of age), Newcastle (at 10 days of age by S/C injection of inactivated vaccine + at 21 days by eye drops), and Infectious Bronchitis (IB) (on the 12th and 20th days of age in drinking water). Throughout the study, they had unrestricted access to food and water. From day 1 to 21 (brooding stage), the birds were fed a starter diet with 23% crude protein (CP) and 3025 kcal/kg metabolizable energy (ME). During the subsequent fattening phase (days 22–37), they received a finisher diet containing 21% CP and 3095 kcal/kg ME (Table 1).

**Table 1:** Composition and calculated analysis of broiler starter and finisher diets

Ingredients, %	Starter diet	Finisher diet
Yellow corn	56.00	61.50
Soybean meal, 44%	28.10	24.87
Corn gluten meal	10.00	7.20
Soybean oil	2.00	2.50
Dicalcium phosphate	1.70	1.70
Limestone	1.45	1.48
NaCl	0.40	0.40
L-lysine	0.05	0.05
Minerals and vitamins premix*	0.30	0.30
Total	100	100
**Calculated analysis**		
Crude protein, %	23.14	21.01
Metabolizable energy, Kcal/kg	3025	3095
Ether extract, %	2.85	2.90
Crude fiber, %	3.51	3.34
Calcium, %	0.97	0.94
Available phosphorus, %	0.47	0.43
Lysine, %	0.98	0.96
Methionine, %	0.70	0.63

* Each 1 kg contains trace minerals premix: 149.60 mg Mn, 16.50 mg Fe, 1.70 mg Cu, 125.40 mg Zn, 0.25 mg Se, 1.05 mg and vitamins premix: 11.023 IU vitamin A, 46.00 IU vitamin E, 3858 IU vitamin D_3_, 1.47 mg menadione, 2.94 mg thiamine, 5.85 mg riboflavin, 20.21 mg pantothenic acid, 0.55 mg biotin, 1.75 mg folic acid, 478.00 mg choline, 16.50 µg vitamin B12, 45.93 mg niacin, and 7.17 mg pyridoxine. A basal diet, according to the National Research Council (1994), was formulated

### Data collection

#### Water analysis

Tap and ionized water samples were collected in triplicate from plastic tanks at the beginning of the experiment, on day 21, and at the end of the experiment. To assess the chemical and microbiological properties of the water, we used a pH meter and a multifunction water meter (DULVAN 7 in 1 Tester Pen, China). We also conducted a phytoplankton examination and a plate count.

#### Performance

Chicks were individually weighed on day 1. The body weight (BW) was recorded at different ages from 60 randomly collected birds in each experimental group, while feed intake (FI) was measured from the six replicates of each treatment. The feed conversion ratio (FCR) was calculated by dividing the total mass of feed intake by the total mass of weight gained. Additionally, the water consumption (WC) and mortality rate of the chicks were determined at the end of the experiment.

#### Biochemical indices

Following a 37-day experimental period, blood samples were collected from forty-eight unsexed birds in each experimental group for subsequent biochemical examinations. Globulin concentration was calculated by subtracting the albumin value from the total protein value, which were both measured according to the manufacturer’s instructions. Plasma triiodothyronine (T_3_) levels were assessed using radioimmunoassay (RIA) kits (Diagnostic Systems Laboratories, USA). Plasma total antioxidant capacity (TAC) was determined according to Benzie and Strain (1996), and reactive oxygen species (ROS) levels were measured in serum using a ROS detection ELISA kit (MBS2801900, USA).

#### Assessment of DNA damage - Alkaline single cell gel electrophoresis (Comet assay - SCGE)

An alkaline comet assay was used to evaluate the genotoxic effect of ionized water in chickens, following the method described by Singh (2000). Blood samples were collected in heparinized tubes and processed immediately. Samples were analyzed in quintuplicate and initially centrifuged at 10,000 rpm. Completely coverslipped microscope slides were coated with 1% normal melting point (NMP) agarose and allowed to polymerize at room temperature. After solidification, the gel was scraped off the slide. The slides were then coated with 0.6% NMP agarose, followed by a second layer containing the whole chicken blood sample mixed with 0.5% low-melting-point (LMP) agarose. Finally, the slides were coated with 0.5% LMP agarose.

The slides were immersed in ice-cold, freshly prepared lysis solution for one hour and then placed in electrophoresis buffer to remove salts. Slides were left in this alkaline buffer for 10 min to allow DNA unwinding and expression at alkali-labile sites. Denaturation and electrophoresis were performed at 4 °C under dim light at 25 V (300 mA). After electrophoresis, the slides were washed three times with buffer to remove excess alkali and detergents. Each slide was stained with ethidium bromide for 10 min and then coverslipped.

Slides were examined using a fluorescence microscope at 250× magnification (Olympus BX41, Japan) and a computer-based image analysis system. A well-trained grader scored 100 randomly selected, non-overlapping comets per sample in a single-blind method. DNA damage was measured by tail length and tail moment, calculated as the product of tail length and tail DNA. Visual analysis was used to detect large-scale damage in comets. Comets were scored individually on a scale of 0–4 based on DNA fragments in their tails. At least 100 cells in each group were scored, following the suggestion of Horvatovich et al. (2013).

#### Gene expression analysis

For gene expression analysis, total RNA was extracted from blood samples using Trizol reagent (GENEzol Reagent, Geneaid, New Taipei, Taiwan) according to the manufacturer’s protocol. Then, cDNA was synthesized from the extracted RNA using reverse transcriptase (Enzynomics, Daejeon, Republic of Korea). Briefly, a 20 µL reverse transcription reaction was prepared by combining 3 µl of total RNA, 0.5 µl of oligo dT primer, 2 µl of dNTPs, 0.5 µl of reverse transcriptase enzyme, 2 µl of reverse transcriptase buffer, and 12 µl of RNase-free water. The reaction was incubated at 37 °C for 2 h, followed by 20 min, incubation at 65 °C, and then cooled at 4 °C for 10 min. Relative gene expression was assessed by q-PCR using the SYBR Green (Enzynomics, Daejeon, Republic of Korea) and gene-specific primers (Table 2). PCR conditions for growth hormone genes were 95 °C for 15 min, and 40 cycles of 95 °C for 30 s, 58 °C for 30 s, 72 °C for 30 s. The qPCR program for water consumption genes was optimized at 50 °C for 120 s, 95 °C for 120 s, and 40 cycles of 95 °C for 15 s, 55 °C for 15 s, and 72 °C for 60 s. Relative gene expression levels were calculated using the 2^−ΔΔct^ method, with GAPDH serving as an endogenous control for normalization.

**Table 2:** Oligonucleotide primer pairs used for gene expression study

Gene	Primer sequence (5′–3′)	Annealing Temperature	Accession Number
Insulin-like growth factor 1 (*IGF-1*)	F: 5’ GATGCTCTTCAGTTCGTATG 3’ R: 5′ TACATCTCCAGCCTCCTC 3′	58 °C	NM_0010043
Chicken Growth hormone (*GH*)	F: 5’ CACCACAGCTAGAGACCCACATC3’ R: 5′ CCCACCGGCTCAAACTGC 3′	58 °C	HE608816
Aquaporin1 (*AQP1*)	F: 5′ AAGTGAGATTGAAGAGCAGTAG 3′ R: 5′ GAACAGCCACAGGAACAA 3′	55 °C	NM_001039453
Calcium-sensing receptor (*CaSR*)	F: 5′ CCTGAGGATTACTGGTCTAATG 3′ R: 5′ GCACAGCAAAGAGAGTTAAAG 3′	55 °C	XM_416491
GAPDH^*^	F: 5′ ATCAAGTGGGGTGATGCTGGT 3′ R: 5′ CCTGCTTCACCACCTTCTTGA 3′	Housekeeping gene	

^*^Glyceraldehyde-3-phosphate Dehydrogenase

### Statistical analysis

Data were analyzed using a one-way ANOVA under a completely randomized design (CRD) with the general linear model (GLM) procedure in SAS (Version 15.1, 2018). Means are presented with their standard errors. Tukey’s test was used for post-hoc comparisons of significant group differences (*p* ≤ 0.05). Relative gene expression was calculated according to Livak and Schmittgen (2001).

## Results

### Water analysis

Table 3 presents chemical and microbiological characteristics of broilers’ drinking water. Significant differences (*p* ≤ 0.05) were observed between the two types of drinking water (tap and ionized water). The ionized water showed lower levels of TDS, algal total count (ATC), and heterotrophic plate count (HPC) than the control. Ionized water also exhibited higher pH and lower ORP values.

**Table 3:** Analysis of drinking water (tap and ionized) used in this study (mean ± SE)

Parameters	Tap water	Ionized water	*P*-value
pH	7.10 ^b^ ±0.01	7.25 ^a^ ±0.01	0.035
ORP (mV)	680 ^a^ ±0.02	−200 ^b^ ±0.02	0.001
Salinity (%)	0.03 ±0.00	0.04 ±0.00	0.163
TDS (ppm)	358 ^a^ ±0.03	344 ^b^ ±0.03	0.040
ATC (unit/mL)	54 ^a^ ±0.01	17 ^b^ ±0.01	0.001
HPC (Cfu/1 mL)	3 ^a^ ±0.00	1 ^b^ ±0.00	0.001
Na^+^ (meq/L)	2.9 ^b^ ±0.01	3.3 ^a^ ±0.01	0.045
K^+^ (meq/L)	0.3 ±0.00	0.3 ±0.00	0.538
Ca^2+^ (meq/L)	1.1 ^b^ ±0.00	1.4 ^a^ ±0.00	0.022
Cl^–^ (meq/L)	2.3 ^b^ ±0.01	2.7 ^a^ ±0.01	0.048
HCO_3_^−^ (meq/L)	2.0 ±0.00	2.1 ±0.00	0.059
CO_3_^2−^ (meq/L)	0.0 ±0.00	0.0 ±0.00	0.717
SO_4_^2−^ (meq/L)	1.1 ±0.00	1.0 ±0.00	0.065

^a, b^ Means having different letters in the same row are significantly different (*p* ≤ 0.05). TDS = total dissolved solids. ATC = algal total count. HPC = heterotrophic plate count. SE: Standard error

### Productive performance

According to Table 4, ionized drinking water significantly (*p* ≤ 0.05) improved the chicks’ performance. The treated chick group (T) had higher BW (at 21 and 37 days of age) and WC, as well as a lower mortality rate than the C group, with significant (*p* ≤ 0.05) different percentages of 7.19, 3.72, 12.23, and 38.90%, respectively.

**Table 4:** The effect of ionized drinking water on the broiler performance (mean ± SE)

Traits	C	T	*P*-value
Body weight^1^ (g)			
1 d	39.41 ± 0.13	39.60 ± 0.15	0.332
21 d	744.50 ^b^ ± 3.02	798.02 ^a^ ± 3.31	0.001
37 d	2079.88 ^b^ ± 5.38	2157.31 ^a^ ± 6.20	0.001
Feed intake^2^ (g/bird)			
1–21 d	931.35 ± 8.20	946.03 ± 7.17	0.063
22–37 d	2292.60 ± 12.11	2305.43 ± 12.98	0.080
1–37 d	3223.95 ± 16.60	3251.46 ± 15.09	0.079
Feed conversion ratio (g: g)			
1–21 d	1.25 ^a^ ± 0.05	1.19 ^b^ ± 0.03	0.023
22–37 d	1.72 ± 0.13	1.70 ± 0.12	0.071
1–37 d	1.55 ^a^ ± 0.03	1.51 ^b^ ± 0.02	0.034
Total water consumption (L)	5.64 ^b^ ± 1.07	6.33 ^a^ ± 1.12	0.023
Total mortality (%)	4.01 ^a^ ± 0.13	2.45 ^b^ ± 0.13	0.006

^a, b^ Means having different letters in the same row are significantly different (*p* ≤ 0.05)

C: Tap water (*n* = 300 chicks), T: Ionized water (*n* = 300 chicks), SE: Standard error. ^1^*n* = 60 chicks/experimental group. ^2^*n* = 6 (replicate’s means of each treatment)

### Biochemical indices

According to the results of Table 5, ionized drinking water can affect some broiler chicks’ blood biochemical parameters within the normal range. The T group showed significantly (*p* ≤ 0.05) higher levels of total protein, globulin, TAC, and T_3_ compared to the C group. They also exhibited lower (*p* ≤ 0.01) ROS levels than the C group.

**Table 5:** The effect of ionized drinking water on the blood biochemical parameters of broiler chicks (mean ± SE)

Traits^1^	C	T	*P*-Value
Total protein (g/dL)	3.50 ^b^ ± 0.11	4.71 ^a^ ± 0.07	0.030
Albumin (g/dL)	2.16 ^b^ ± 0.04	2.52 ^a^ ± 0.05	0.042
Globulin (g/dL)	1.34 ^b^ ± 0.05	2.19 ^a^ ± 0.05	0.029
T_3_ (ng/dL)	2.91 ^b^ ± 0.07	3.82 ^a^ ± 0.09	0.002
TAC (mmol/l)	1.15 ^b^ ± 0.03	1.70 ^a^ ± 0.05	0.002
ROS (pg/mL)	135.00 ^a^ ± 11.53	98.01 ^b^ ± 12.10	0.001

^a, b^ Means having different letters in the same row are significantly different (*p* ≤ 0.05)

C: Tap water, T: Ionized water, SE: Standard error. ^1^*n* = 48 samples/experimental group

T_3_ = triiodothyronine. TAC = total antioxidant capacity. ROS = reactive oxygen species

### Comet assay (Single-cell gel electrophoresis)

**Fig. 1:**
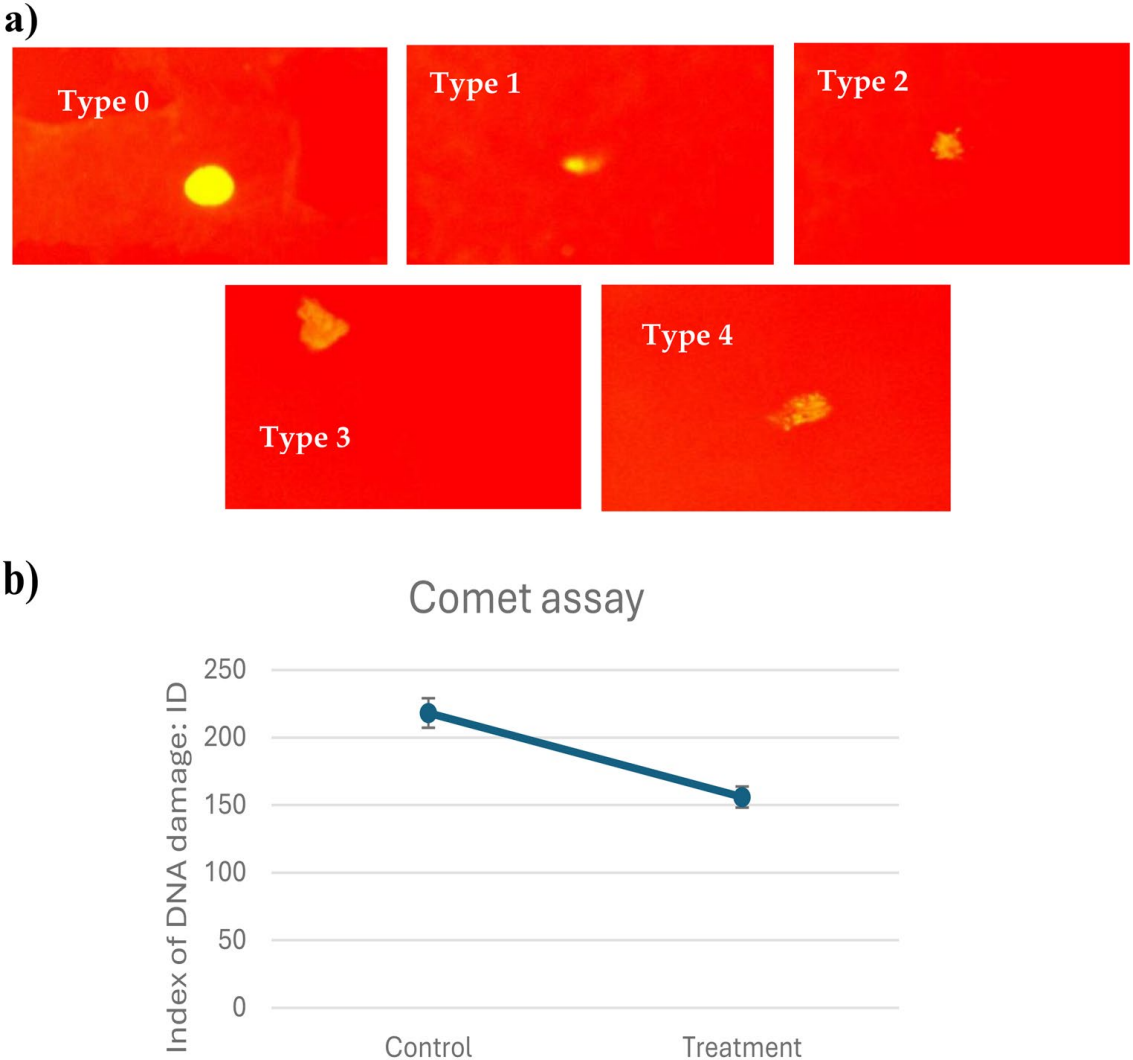
Comet assay analysis of using ionized drinking water for chickens. (**a**): Cell shape under the fluorescence microscope after staining with ethidium bromide showed the different types of DNA damage, (**b**): Graph of DNA-damaged index. Differences showing statistical significance at *P*-value < 0.001 compared to the control group

The comet assay was performed to assess DNA damage after exposure to ionized water (Fig. 1). A significant reduction in DNA damage was observed in the treatment group compared to the control group. Specifically, the mean comet tail moment in the control group was 218 ± 12, while in the treatment group, it was 156 ± 5. This value represents a 28.44% reduction in DNA damage in the treatment group. The difference was statistically significant (*t* (4) = 4.337, *p* = 0.012). These results indicate that the ionized water treatment was effective in reducing DNA damage.

### Gene expression analysis (qRT-PCR)

**Fig. 2:**
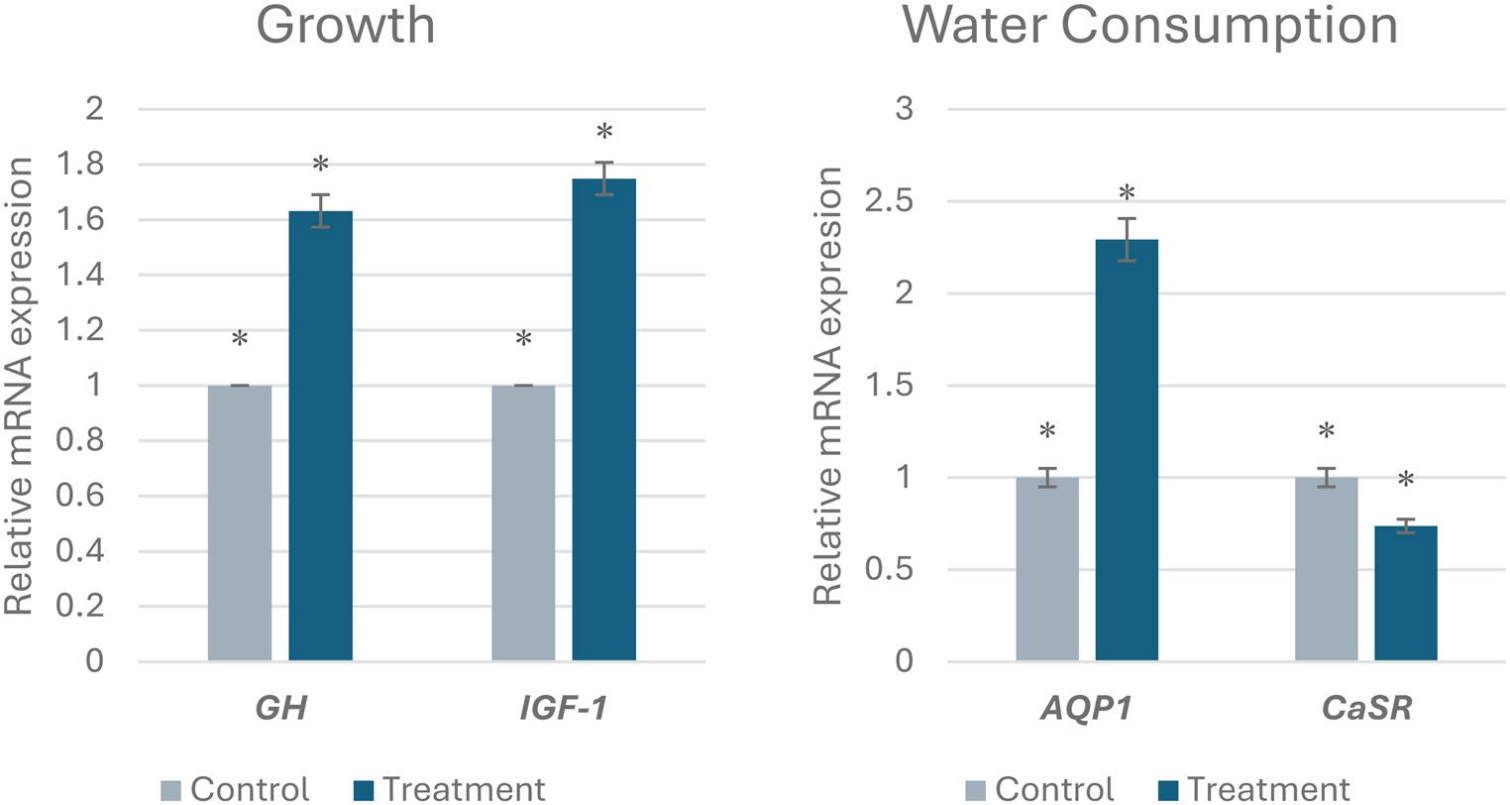
The effect of ionized water on the mRNA expression of *GH*, *IGF*, *AQP1*, and *CaSR* in chickens. *Differences that show statistical significance (*P* < 0.05) compared to the control group

*IGF-1* and *GH* mRNA expression were evaluated relative to growth hormone expression in chickens from the control and ionized water treatment groups (Fig. 2). The expression of both *GH* and *IGF* was significantly upregulated in the treatment group compared to the control group. Specifically, *GH* expression in the treatment group showed a 1.63-fold increase compared to the control group (*p* = 0.03956). Similarly, *IGF* expression in the treatment group showed a 1.75-fold increase compared to the control group (*p* = 0.0049). These results indicate that treatment significantly increased the expression of *GH* and *IGF*.

The effect of treatment on water consumption gene expression was assessed by qRT-PCR. *AQP1* expression. It was significantly upregulated in the treatment group (fold change = 2.29) compared to the control group (*p* = 0.041). In contrast, *CaSR* expression was significantly downregulated in the treatment group (fold change = 0.74) compared to the control group (*p* = 0.0001). These results indicate that the treatment differentially affects the expression of *AQP1* and *CaSR*, resulting in increased *AQP1* expression and decreased *CaSR* expression.

## Discussion

### Water analysis

Poultry farming faces significant hurdles due to poor water quality, driving a shift towards treated, functional water with distinct advantages. Several specialized methods exist for producing this type of water, including magnetization and electrolysis. Applying an electric field to water can modify its chemical properties, leading to increased pH, TAC, and levels of essential minerals like calcium (Jassim and Aqeel 2017; Mohamed et al. 2025). Additionally, this process results in smaller clusters of water molecules compared to ordinary water, potentially improving cellular water absorption (Ezzat et al. 2017; Mohamed et al. 2025). Electrolyzing drinking water can also enhance water quality by increasing molecular ionization and fluidity, in addition to beneficial water with a negative ORP, indicating antioxidant properties and reduced bacterial and algae content compared to tap water (Shihab et al. 2019; Salama and Alaa 2024).

Given that high-quality drinking water is crucial for maintaining optimal biological functions, enhancing health, reducing stress, and supporting productivity in animals, and the current gap in understanding the specific effects of ionized alkaline water on poultry productivity and DNA damage, this study sought to determine the impact of drinking ionized water on the growth performance and DNA integrity of broiler chicks.

The control group’s chicks received tap water, which served as the standard drinking water source for comparison. The quality of the water can be described by several chemical and physical analysis parameters. The water analysis results in Table 3 indicate that the ionization process of broiler drinking water has demonstrated positive effects on key water quality parameters. Specifically, ionization influences the ORP, often resulting in a more negative value, which may suggest enhanced antioxidant properties. Furthermore, it has been shown to contribute to a more balanced pH level, which is crucial for maintaining broiler health and digestive function. Additionally, water ionization can lead to a reduction in TDS, indicating a decrease in the concentration of dissolved substances, which collectively contributes to improved overall water quality for broiler chickens. These findings also link to cellular health because the negative ORP of alkaline drinking water may help neutralize harmful free radicals by donating electrons. Free radicals are unstable molecules produced by the body that can damage proteins, fats, and DNA (Mishra and Jha 2019; Oke et al. 2024). Moreover, a notable distinction was observed between tap water and ionized water regarding ATC and HPC levels. The ionized water exhibited lower levels, which may contribute to the enhancement of some productive traits.

### Performance

Slightly alkaline ionized drinking water (7.25) with a negative ORP value (-200 mV) has been shown to improve chicks’ performance, including BW, FCR, WC, and lower mortality rates (Table 4). It has excessive oxygen in the form of OH^−^ instead of O_2_, which is more stable and inhibits the formation of scale (Salama and Alaa 2024; Mohamed et al. 2025). Maintaining the right electrolyte balance in broiler drinking water is key for regulating birds’ blood and fluid levels (Sayed and Downing 2015). Furthermore, alkaline ionized water positively influences birds’ gut pH values, gut microflora balance, and the metabolic processes (Mohamed et al. 2025). It also acts as an antioxidant, which can help neutralize free radicals and reduce oxidative stress (Delos Reyes et al. 2021). These effects result in improved BW and FCR.

The observed higher WC in the T group compared to the C could stem from the palatable nature and elevated sodium content (salinity %) of the ionized drinking water, contrasting with tap water. Likewise, Abbas et al. (2008) suggested that lower TDS can enhance palatability and thus increase water consumption. Aligning with these findings, Ebrahimi et al. (2024) underscored the importance of water treatment and quality enhancement in poultry, as the water’s physicochemical properties influence both consumption and bird performance. Furthermore, Khan et al. (2013) demonstrated that slightly alkaline water (around pH 7.3) significantly improved body weight gain and feed conversion efficiency in birds compared to those receiving ordinary, near-neutral pH water.

Contaminants like bacteria, algae, and chemicals pose a significant threat to water quality on poultry farms. Therefore, routine testing of water quality parameters, particularly microbial content, is crucial for identifying and resolving potential issues that could negatively affect poultry health and productivity. However, to reduce the risk of waterborne diseases and ensure clean, safe drinking water for birds, suitable treatment methods like ionization can be implemented. Similarly, exposing water to an electric field can alter its components, including the microbial load, with the resulting effects varying based on the electric field’s strength and exposure duration. From this standpoint, the most significant advantage of using ionized water is to reduce pathogenic bacteria by affecting their membranes and the unavailability of needed chemicals (Rahman et al. 2023; Mohamed et al. 2025). According to the results of Table 4, there are significant improvements in broiler’s productive traits. These improvements can be due to the beneficial influence of ionized water on chicks’ intestinal health and gut integrity by modulating the intestinal environment and promoting gut microflora competitive exclusion.

### Biochemical indices

Blood biochemical parameters monitor physiological activity changes and reflect animal health and production (Elsayed et al. 2024). Slightly alkaline drinking water with negative ORP values has also been shown to improve chicks’ oxidative status (increases TAC and decreases ROS), and some blood biochemical traits, such as total protein, globulin, T_3_, compared to the control (Table 5). Notably, the provision of alkaline ionized water appears to offer advantages for poultry physiology and productivity. Ezzat et al. (2023) found that it elevated hemoglobin and hematocrit levels in quail. Likewise, Jassim and Aqeel (2017) reported enhanced productive performance in birds receiving alkaline drinking water, noting improvements in blood biochemical traits like total protein and cholesterol. In the current study, the observed slight increase in growth-related hormones, such as T_3_, might directly contribute to productive performance enhancement. Mahbuba (2007) suggested this occurs by stimulating the thyroid gland to release thyroxin, subsequently boosting feed intake and improving fat and protein metabolism. Furthermore, McCreery (2003) mentioned that treated drinking water can aid in cell regeneration and enhance ion transport across cell membranes. The resulting increase in final body weights is potentially linked to this improved water quality, leading to better water intake and feed conversion rates. However, it is crucial to note, as Martínez et al. (2021) pointed out, that excessively alkaline drinking water can have negative consequences, adversely affecting the relative weight of immune organs like the spleen, as well as cecal pH and bacterial counts. Consequently, the administration of alkaline water requires careful consideration due to its direct influence on both the health and productivity of broiler chicks.

Management of reactive oxygen species (ROS) levels is critical for maintaining poultry health and optimizing production. In this study, ROS levels were measured in the blood samples of the control and treated chicken groups (Table 5). The T group showed a significant decrease in ROS levels (27.41%) compared to the C group. This result indicates that the ionization treatment of chick drinking water was effective in reducing ROS production, which alters the bird’s overall performance (Mishra and Jha 2019). Hence, ionized alkaline drinking water can act as an antioxidant that scavenges ROS and mitigates oxidative stress. In the same manner, Ezzat et al. (2017) demonstrated that treated alkaline drinking water can mitigate oxidative stress, a significant issue affecting the physiological functions and health of chickens in contemporary production (El-Sabrout et al. 2024), particularly in tropical and subtropical areas.

### Comet assay (Single-cell gel electrophoresis - SCGE)

The comet assay is a widely used and versatile tool for assessing DNA damage in various contexts, including food safety and toxicology (Bivehed et al. 2023). It has proven effective in evaluating the genotoxic effects of different chemicals and contaminants in commercially important poultry species such as chickens and turkeys (Gajski et al. 2019). Sokolovic et al. (2007) demonstrated the genotoxic effects of T-2 toxin in chickens using the SCGE technique, while Braga-Neto et al. (2023) showed the effectiveness of this technique in detecting lead-induced chromosomal damage, revealing a dose-response relationship. In addition to detecting damage, the comet assay can be used to evaluate protective agents. Zimmermann et al. (2015) revealed that β-glucan can attenuate aflatoxin B1-induced genotoxicity in chicken lymphocytes, while Awad et al. (2014) demonstrated the protective effect of a microbial feed additive against deoxynivalenol-induced DNA damage in chickens. Similarly, Da Silva Cardoso et al. (2016) used the comet and micronucleus assays to show that piperine protects chickens against aflatoxin B1-induced DNA damage. In the current study, the comet assay was performed to assess DNA damage after exposing broiler chicks to ionized drinking water for 37 days (marketable age) (Fig. 1). A significant reduction in DNA damage was observed in the treated group compared to the control group. Specifically, the mean comet tail moment in the control group was 218 ± 12, while in the treatment group it was 156 ± 5. This value represents a 28.44% reduction in DNA damage in the treated chicks. This result indicates that the ionization process of drinking water was effective in reducing DNA damage.

### Gene expression analysis (qRT-PCR)

*GH* plays a critical role in the development and metabolism of hepatocytes by stimulating hepatic *IGF-1* secretion, which in turn promotes bone and muscle cell differentiation and proliferation (Kuhn et al. 2002). *IGF-1*, a peptide hormone, mediates many of the growth-promoting activities of *GH* in poultry (Anh et al. 2015). Consistent with these established roles, Saxena et al. (2020) found that nutrition changes upregulated growth-related genes and improved the growth performance of broiler chickens. Our results similarly showed a statistically significant increase in *GH* and *IGF-1* gene expressions. Furthermore, water consumption, which is positively correlated with growth, has likely increased in modern broiler populations compared to previous years due to increasing market weights (Edge et al. 2025). *AQP1* plays a key role in renal water movement and retention (Yang et al. 2001). Additionally, the *CaSR* regulates blood calcium levels; increased calcium binding to the *CaSR* inhibits calcium reabsorption (Canaff and Hendy 2005; Ruat and Traiffort 2013). Aggrey et al. (2023) demonstrated upregulated *AQP1* and *CaSR* expressions in chickens with low relative humidity and water consumption, indicating increased renal water reabsorption and highlighting the importance of these genes in water homeostasis. The current findings further demonstrate that altered water consumption differentially affects *AQP1* and *CaSR* expressions: *AQP1* expression is increased in the treated group while *CaSR* expression is decreased. This suggests that the ionization treatment can affect the expression of genes involved in different biological processes. *AQP1* is a water channel protein; therefore, its upregulation could indicate changes in water balance or fluid transport, while *CaSR* is a calcium-sensing receptor, and its downregulation could indicate changes in calcium homeostasis or related signaling pathways (Aggrey et al. 2023).

Ionized water typically has an altered pH level and ORP compared to regular tap water. The slightly alkaline pH often associated with ionized water can modulate enzyme activity, protein conformation, and receptor binding. All changes can affect the cellular machinery responsible for gene expression (Casey et al. 2010). An environment with a higher reduction potential due to ionized water might affect transcription factor activity or oxidative stress pathways, thereby impacting the expression of genes such as *GH* and *IGF-1*, which are sensitive to cellular metabolic states (Jones and Sies 2015; Priya Dharshini et al. 2020).

Furthermore, the influence of ionized water may stem from its ability to alter mineral solubility and absorption. For instance, if ionized water affects calcium bioavailability, it could directly impact *CaSR* activity and subsequent gene expression. A decrease in *CaSR* expression, as observed, might reflect a physiological adaptation to changes in circulating calcium levels or a direct effect of the ionized water on the cellular environment (Díaz-Soto et al. 2016). Similarly, *AQP1* upregulation could be a compensatory mechanism to maintain water balance in response to altered water properties or increased water consumption induced by the treatment. It is plausible that the ionic composition or subtle structural changes in ionized water facilitate its absorption and distribution within the body, thereby triggering adaptive responses in genes related to water transport and ion homeostasis. Hence, ionized alkaline water could be recommended as the main drinking water source for broiler chickens, potentially enhancing growth performance and oxidative status while reducing bird mortality and DNA damage (Tables 4 and 5, and Figs. 1 and 2).

Based on the previous findings, alkaline ionized water appears to foster a more balanced internal environment in birds, potentially enhancing several physiological functions. The altered molecular structure of ionized alkaline water may improve its absorption in the birds’ intestinal cells. This enhanced absorption could lead to better nutrient utilization from their feed, potentially resulting in increased growth rates and body weights. Moreover, more efficient nutrient use could lower the feed conversion ratio, thus reducing costs. The antioxidant properties of ionized water may also help decrease stress, bolster the birds’ immune systems, lower mortality rates, and improve genomic DNA integrity, which is vital for cell function and survival. Consequently, using green technology like tap water ionization, broiler breeders can ensure their birds receive water that meets their specific needs for optimal growth and improved health without adverse physiological effects. However, further investigation is required into the effects of higher ionization levels on chick health and productivity.

## Conclusion

To the best of our knowledge, this is the first report investigating the effects of ionization treatment of drinking water on broiler chicken growth genes’ expression, oxidative status, and DNA integrity. Based on the current findings, the ionization process improves drinking water quality by influencing water ORP, pH, and TDS. Ionizing drinking water positively affected broilers’ growth, water consumption, and final body weights. It also enhances chicks’ oxidative status and some blood biochemical traits, such as total protein, globulin, T_3_, TAC, as well as lowering ROS levels compared to the control. Furthermore, the current results indicate that the use of ionized drinking water increases *GH*, *IGF-1*, and *AQP1* expression levels while decreasing *CaSR* and DNA damage. Consequently, applying water ionization for broiler chicken drinking water is recommended to enhance animal productivity and health. Further research is warranted to optimize the use of ionized water in poultry farms.

## Data Availability

The supplementary data can be available from the corresponding author upon a reasonable request.

## References

[CR1] Abbas TE, Elzubeir EA, Arabbi OH (2008) Drinking water quality and its effects on broiler chicks performance during winter season. Inter J Poult Sci 7(5):433–436

[CR2] Aggrey SE, Ghareeb AFA, Milfort MC, Ariyo OW, Aryal B, Hartono E, Kwakye J, Sovi S, Hipple SA, Stevenson C, Fuller AL, El Sabry MI, Stino F, Rekaya R (2023) Quantitative and molecular aspects of water intake in meat-type chickens. Poult Sci 102(11):102973 37633082 10.1016/j.psj.2023.102973 PMC10474491

[CR3] Anh NTL, Kunhareang S, Duangjinda M (2015) Association of chicken growth hormones and insulin-like growth factor gene polymorphisms with growth performance and carcass traits in Thai broilers. Asian-Aust J Anim Sci 28(12):1686 10.5713/ajas.15.0028 PMC4647076 26580435

[CR4] Awad WA, Ghareeb K, Dadak A, Hess M, Böhm J (2014) Single and combined effects of Deoxynivalenol Mycotoxin and a microbial feed additive on lymphocyte DNA damage and oxidative stress in broiler chickens. PLoS ONE 9(1):e88028 24498242 10.1371/journal.pone.0088028 PMC3909330

[CR5] Benzie IF, Strain JJ (1996) The ferric reducing ability of plasma (FRAP) as a measure of antioxidant power: the FRAP assay. Anal Biochem 239:70–76 8660627 10.1006/abio.1996.0292

[CR6] Bivehed E, Hellman B, Fan Y, Haglöf J, Buratovic S (2023) DNA integrity under alkaline conditions: an investigation of factors affecting the comet assay. Mutat Research/Genetic Toxicol Environ Mutagen 891:503680 10.1016/j.mrgentox.2023.503680 37770137

[CR7] Braga-Neto JT, Tozetto SO, Oliveira FS, Conceição TA, Santos WPC, Fernandes MS, Baliza MD (2023) Comet assay to evaluate chromosomal changes in chickens (*Gallus Gallus domesticus*) contaminated by lead in a City in Bahia. Braz J Biol 83:e274806 38126633 10.1590/1519-6984.274806

[CR8] Canaff L, Hendy GN (2005) Calcium-sensing receptor gene transcription is up-regulated by the Proinflammatory cytokine, interleukin-1beta. Role of the NF-kappaB PATHWAY and kappab elements. J Biol Chem 280(14):14177–14188 15684428 10.1074/jbc.M408587200

[CR9] Casey JR, Grinstein S, Orlowski J (2010) Sensors and regulators of intracellular pH. Nature reviews. Mol Cell Biol 11(1):50–61 10.1038/nrm2820 19997129

[CR10] Da Silva Cardoso V, Vermelho AB, Ribeiro de Lima CA, Mendes de Oliveira J, de Freire ME, Pinto, da Silva LH, Direito GM, Miranda Danelli MDG (2016) Antigenotoxic effect of piperine in broiler chickens intoxicated with Aflatoxin B1. Toxins 8(11):316 10.3390/toxins8110316 PMC5127113 27809242

[CR11] Delos Reyes FSLG, Mamaril ACC, Matias TJP, Tronco MKV, Samson GR, Javier ND, Fadriquela A, Antonio JM, Sajo MEJV (2021) The search for the elixir of life: on the therapeutic potential of alkaline reduced water in metabolic syndromes. Processes 9:1876

[CR12] Díaz-Soto G, Rocher A, García-Rodríguez C, Núñez L, Villalobos C (2016) The calcium-sensing receptor in health and disease. Inter Rev Cell Mol Biol 327:321–369 10.1016/bs.ircmb.2016.05.004 27692178

[CR13] Ebrahimi NA, Nobakht A, İnci H, Palangi V, Suplata M, Lackner M (2024) Drinking water quality management for broiler performance and carcass characteristics. World 5:952–961

[CR14] Edge CM, Davis JD, Purswell JL, Campbell JC, Batchelor WD, Linhoss JE (2025) Water consumption trends for commercial broilers grown to nine weeks. J Appl Poult Res 34(2):100511

[CR15] El-Sabrout K, Landolfi S, Ciani F (2024) Feed additives and enrichment materials to reduce chicken stress, maximize productivity, and improve welfare. Vet World 17:2044–2052 39507789 10.14202/vetworld.2024.2044-2052 PMC11536731

[CR16] Elsayed M, Soliman F, Elghalid O, El-Sabrout K (2024) Using different cage enrichments to improve rabbits’ performance, behavior, and welfare. Animals 14:2271 39123797 10.3390/ani14152271 PMC11310941

[CR18] Ezzat HN, Shihab IM, Hussein MA (2017) Effects of ionized water on certain egg quality traits and the levels of proteins and enzymes in the blood of the Japanese quail *Coturnix Japonica*. Inter J Poult Sci 16:69–80

[CR17] Ezzat HN, Hussein MA, Shihab IM, Al-Qazzaz MF (2023) Effect of drinking ionized water on histological changes, bacteria count, and some hematological parameters of Japanese quail. Iraqi J Agric Sci 54:464–471

[CR19] Gajski G, Žegura B, Ladeira C, Novak M, Sramkova M, Pourrut B, Del Bo’ C, Milić M, Bjerve Gutzkow K, Costa S, Dusinska M, Brunborg G, Collins A (2019) The comet assay in animal models: from Bugs to whales – (Part 2 Vertebrates). Mutat Research/Reviews Mutat Res 781:130–164 10.1016/j.mrrev.2019.04.002 31416573

[CR20] Horvatovich K, Hafner D, Bodnár Z, Berta G, Hancz Cs., Dutton M, Kovács M (2013) Dose-related genotoxic effect of T-2 toxin measured by comet assay using peripheral blood mononuclear cells of healthy pigs. Acta Vet Hung 61:175–186 23661386 10.1556/AVet.2013.010

[CR21] Hu D, Kabayama S, Watanabe Y, Cui Y (2024) Health benefits of electrolyzed hydrogen water: antioxidant and anti-inflammatory effects in living organisms. Antioxidants 13(3):313 38539846 10.3390/antiox13030313 PMC10967432

[CR22] Jassim EQ, Aqeel CH (2017) Effect of alkaline water and/or magnetic water on some physiological characteristics in broiler chicken. J Entomol Zool Stud 5:1643–1647

[CR23] Jones DP, Sies H (2015) The redox code. Antioxid Redox Signal 23(9):734–746 25891126 10.1089/ars.2015.6247 PMC4580308

[CR24] Khan S, Sultan A, Muhammad A, Imtiaz N, Mobashar M, Khan H, Saleem M, Inam M, Rafiullah (2013) Lower illeal microflora and growth performance of broilers supplemented with organic acid blend (Aciflex^®^) during starter phase. Greener J Agric Sci 3:794–800

[CR25] Kuhn ER, Vleurick L, Edery M, Decuypere E, Darras VM (2002) Internalization of the chicken growth hormone receptor complex and its effect on biological functions. Comp Biochem Physiol B: Biochem Mol Biol 132(1):299–308 11997231 10.1016/s1096-4959(02)00037-4

[CR26] Livak KJ, Schmittgen TD (2001) Analysis of relative gene expression data using real-time quantitative PCR and the 2^–∆∆CT^ method. Methods 25(4):402–408 11846609 10.1006/meth.2001.1262

[CR27] Mahbuba AM Effect of magnetic technology of water treatment on productive and physiological performance of embryos, broiler, breeders and hatched chicks in different climate. Ph.D., Thesis (2007) Collage of Agriculture-Univ., Baghdad, Iraq

[CR28] Martínez Y, Almendares CI, Hernández CJ, Avellaneda MC, Urquía AM, Valdivié M (2021) Effect of acetic acid and sodium bicarbonate supplemented to drinking water on water quality, growth performance, organ weights, cecal traits and hematological parameters of young broilers. Animals 11:1865 34201537 10.3390/ani11071865 PMC8300354

[CR29] McCreery A (2003) Magnetic water Raising your pH-Life sources. LiveSource, Inc., Paris, France

[CR30] Mishra BK, Jha R (2019) Oxidative stress in the poultry gut: potential challenges and interventions. Front Vet Sci 6:60 30886854 10.3389/fvets.2019.00060 PMC6409315

[CR31] Mohamed A, Khalil M, Soliman F, El-Sabrout K (2025) The effect of drinking ionized water on the productive performance, physiological status, and carcass characteristics of broiler chicks. Animals 15:229 39858229 10.3390/ani15020229 PMC11758290

[CR32] Oke OE, Akosile OA, Oni AI, Opowoye I, Ishola C, Adebiyi J, Odeyemi A, Adjei-Mensah B, Uyanga VA, Abioja M (2024) Oxidative stress in poultry production. Poult Sci 103:104003 39084145 10.1016/j.psj.2024.104003 PMC11341942

[CR33] Priya Dharshini LC, Vishnupriya S, Sakthivel KM, Rasmi RR (2020) Oxidative stress responsive transcription factors in cellular signalling transduction mechanisms. Cell Signal 72:109670 32418887 10.1016/j.cellsig.2020.109670

[CR34] Rahman SM, Islam SM, Kong D, Xi Q, Du Q, Yang Y, Oh D, Wang J, Han R (2023) Controlling microbial population in livestock and poultry industry using electrolyzed water as an emerging technology for ensuring food safety. Food Control 152:109843

[CR35] Ruat M, Traiffort E (2013) Roles of the calcium sensing receptor in the central nervous system. Best Pract Res Clin Endocrinol Metab 27(3):429–442 23856270 10.1016/j.beem.2013.03.001

[CR36] Salama HF, Alaa OA (2024) The role of ionized water as a safe alternative to disinfectants in poultry slaughter houses. Egypt J Anim Health 4:50–60

[CR37] Saxena R, Saxena VK, Tripathi V, Mir NA, Dev K, Begum J, Agarwal R, Goel A (2020) Dynamics of gene expression of hormones involved in the growth of broiler chickens in response to the dietary protein and energy changes. Gen Comp Endocrinol 288:113377 31881203 10.1016/j.ygcen.2019.113377

[CR38] Sayed MAM, Downing J (2015) Effects of dietary electrolyte balance and addition of electrolyte–betaine supplements in feed or water on performance, acid–base balance and water retention in heat-stressed broilers. Br Poult Sci 56:195–209 25558900 10.1080/00071668.2014.995594

[CR39] Shihab IM, Ezzat HN, Hussein MA (2019) Effect of using ionized water on some productive and physiological performance of Japanese quails. Poult Sci 98(10):5146–5151 30726967 10.3382/ps/pey590

[CR40] Singh NP (2000) Microgels for Estimation of DNA strand breaks, DNA protein crosslinks and apoptosis. Mutat Res 455:111–127 11113471 10.1016/s0027-5107(00)00075-0

[CR41] Sokolovic M, Garaj-Vrhovac V, Ramic S, Simpraga B (2007) Chicken nucleated blood cells as a cellular model for genotoxicity testing using the comet assay. Food Chem Toxicol 45(11):2165–2170 17618029 10.1016/j.fct.2007.05.013

[CR42] Yang B, Ma T, Verkman AS (2001) Erythrocyte water permeability and renal function in double knockout mice lacking aquaporin-1 and aquaporin-3. J Biol Chem 276:624–628 11035042 10.1074/jbc.M008664200

[CR43] Zimmermann CEP, Cruz IBM, Cadoná FC, Machado AK, Assmann C, Schlemmer KB, Zanette RA, Leal DBR, Santurio JM (2015) Cytoprotective and genoprotective effects of β-glucans against aflatoxin B1-induced DNA damage in broiler chicken lymphocytes. Toxicol Vitro 29(3):538–543 10.1016/j.tiv.2015.01.005 25615424

